# Content validation of the SF-36v2® Health Survey Acute for use in hypoparathyroidism

**DOI:** 10.1007/s11136-023-03352-x

**Published:** 2023-02-09

**Authors:** Meryl Brod, Laura Tesler Waldman, Aimee D. Shu, Alden Smith

**Affiliations:** 1 The Brod Group, 219 Julia Ave, Mill Valley, CA 94941 USA; 2 Ascendis Pharma, Inc, 1000 Page Mill Rd, Palo Alto, CA 94301 USA

**Keywords:** Hypoparathyroidism, SF-36v2® Health Survey Acute, Content validation, Adults

## Abstract

**Purpose:**

The purpose of this study was to conduct cognitive debriefing (CD) interviews with adults diagnosed with chronic hypoparathyroidism (HP) to assess the content validity of the SF-36v2® Health Survey Acute (SF-36v2) measure in this population.

**Methods:**

CD interviews were conducted with adults with HP in the United States (US). Interviews were conducted by a trained moderator using a semi-structured interview guide, employing a think-aloud method in conjunction with verbal probing. Participants were asked whether each item was understandable, relevant, important, and sensitive to change in relation to HP. Additionally, comprehension of instructions, response options, and the appropriateness of a 1-week recall period was assessed.

**Results:**

Sixteen adults with HP participated in individual CD telephone interviews. All items in the SF-36v2 were reported to be understood, relevant, important, and sensitive to change by at least half, and in most cases, by a strong majority of study participants. Most of the study sample confirmed comprehension of the instructions and the entire sample understood all response options.

**Conclusion:**

The study findings show that the items in the SF-36v2® are applicable to adults with HP. The overall high levels of endorsement of items provide strong evidence of the measure’s content validity for this population. The SF-36v2 is therefore recommended for usage in clinical trials examining adults with HP, although it is recommended that this generic measure be supplemented with disease-specific instruments such as the recently developed Hypoparathyroidism Patient Experience Scale—Symptom (HPES-Symptom) and Hypoparathyroidism Patient Experience Scale—Impact (HPES-Impact) measures.

## Background

Chronic hypoparathyroidism (HP) is a rare endocrine disease requiring conventional therapy of oral calcium supplements and active vitamin D to sustain normal serum levels [1, 2]. Chronic HP is associated with significant physical, psychological, and cognitive symptoms despite use of conventional therapy [1]. Prior studies have found that patients with HP who are on conventional therapy report significant symptoms, such as fatigue, muscle spasms, paresthesia, anxiety, depression, and cognitive dysfunction/ “brain fog”, which may be indicative of a reduced quality of life despite control of biochemical parameters [3–9].

The SF-36v2® Health Survey Acute (herein referred to as the “SF-36v2”) is a validated measure that has previously been used in several studies examining burden of illness and impact of treatment experienced by adult patients with HP [3–5, 10, 11]. However, to the best of our knowledge, the content validity of this measure has not been formally assessed for this population. Therefore, the purpose of this project was to conduct cognitive debriefing (CD) interviews with adults who have HP in order to assess the content validity of the SF-36v2 measure in this population.

The study was approved by Institutional Review Board (IRB) WCG IRB, located in Puyallup, Washington, US [AS-HP-SF-36 CD 2021; IRB Number: 20213924]. Informed consent was obtained from all study participants.

## Methodology

The CD interview process was developed in accordance with the United States (US) Food and Drug Administration guidance on best practices for establishing the content validity of patient-reported outcome (PRO) instruments for use in clinical trials [12], and best practices for the conduct of cognitive debriefing interviews [13, 14]. The methodological details of the study were reviewed using the COREQ checklist and the design details met all applicable checklist criteria.

CD interviews were conducted with 18 adults with HP in the US between September and October 2021. Individuals with HP were recruited from a database of people who had participated in previous research conducted by The Brod Group (TBG) who had been recruited via a national HP organization and had given permission to be contacted about future studies, and were contacted by TBG research staff to assess their interest and verbally confirm eligibility to participate in the present study. Participants in prior studies had received an honorarium approved by the ethics review board. To be eligible for the study, participants were required to be at least 18 years old; be able to read, write, and speak English; have a diagnosis of HP for at least 6 months; be stable on conventional therapy for at least 3 months, which was defined as experiencing infrequent severe hypo- or hypercalcemia with high-normal to elevated urine calcium excretion (not precluding occasional [≤ 2/week] rescue doses of active vitamin D and/or calcium for symptomatic hypocalcemia); and have a BMI between 17 and 40 kg/m^2^. Exclusion criteria were having impaired renal function, defined as having stage 4 or 5 chronic kidney disease (eGFR ≤ 30 ml/min/1.73 m^2^); or a cognitive impairment or any other medical condition, including psychiatric disorders (e.g., bipolar disorder, schizophrenia), which would impact their ability to participate in a one-time, one-on-one telephone interview about their experience with HP.

All interviews were conducted and coded by a female, PhD level qualitative researcher/trained moderator (second author) familiar with HP who followed a semi-structured interview guide which was used to ask participants questions employing a think-aloud method in conjunction with verbal probing as needed [13, 14]. All interviews were conducted by telephone and lasted approximately 1 h. At the beginning of the interview, the interviewer established rapport with participants by introducing herself, and discussing who she worked for, the study’s purpose, which was to ‘test’ the survey on health in general to make sure that it was appropriate and relevant, and that the participant’s role in the study was to help make sure that everything in the survey was relevant and appropriate to ask. Additionally, an opportunity for the participant to ask questions about the informed consent form was provided.

The SF-36v2 is a 36-item generic, multipurpose health survey with questions that yield an 8-scale profile of functional health and well-being, as well as 2 psychometrically based physical and mental health summary scores and a preference-based health utility index [15]. Participants were emailed the survey and asked to print out and complete the survey 24–48 h prior to the interview and have the completed survey on hand for the call. Then during the telephone interview, participants were verbally asked questions based on the discussion guide regarding (1) their comprehension of instructions and items such as *What are the instructions asking you to do/think about?* and *In your own words, what does the question mean to you?,* (2) item relevance such as *Was the question about something relevant to your experience with hypoparathyroidism?,* (3) item importance such as *Is this question about an important aspect of how HP affects your functioning and well-being?,* and (4) item sensitivity to change such as *Do you think if your HP improved, that it would change how you answered this question?* At the end of each item or set of items with a unique response option cluster, participants were asked whether the response choices made sense and finally, at the end of the interview, participants were asked questions about whether the measure’s recall period was appropriate and if they were accurately able to remember their experiences over the past week.

Interviews were conducted in blocks of 4 participants each. In between each block, the findings were documented in an item tracking matrix and reviewed by the study team to determine whether any significant issues had been raised by participants about the measure’s instructions, items, and/or response options. All interviews were audio-recorded and transcribed.

## Data analysis

Participants’ responses, comments, and suggested changes were organized and compiled into a detailed CD tracking matrix, recorded on an Excel spreadsheet, which was initially based on the interviewer’s notes recorded during and/or right after the interview, and subsequently confirmed and supplemented through a review of the full interview transcripts. These findings were iteratively reviewed by the research team to identify potential signals of true problems with comprehension or content for this population vs. minor individual and/or style-based preferences, following the similar procedures used for other SF-36v2 content validation studies [16–18].

## Results

Fifty-two potential participants were contacted via email regarding their interest in the study. Twelve of the 52 individuals did not respond to our outreach. Of those who responded (*n* = 40), none refused participation. Potential participants were screened for eligibility and of those eligible, 18 were selected to be interviewed based on characteristics which would allow for as diverse a sample as possible (16 of whom had participated in previous research conducted by TBG). Two participants were excluded due to reporting during their interview that they had, for the past several years, not had any symptoms from their HP resulting in an analysis sample of 16.

### Participant characteristics

Self-reported, detailed demographic, health, disease, and treatment characteristics for the 16 participants who were included in the analysis are shown in Table 1.

**Table 1:** Participant demographic, general health, disease, and treatment characteristics

Participant characteristics	Total (*n* = 16)
Gender*, n*(%)	
Female	14 (87.5)
Male	2 (12.5)
Age	
Mean (SD)	51.5 (11.7)
Median	54.5
(Range)	(29–69)
Marital status, *n*(%)	
Married	9 (56.2)
Partnered	3 (18.8)
Divorced	4 (25.0)
Race/ethnicity^a^, *n*(%)	
White/Caucasian	15 (93.8)
Hispanic/Chicano/Latino	1 (6.3)
Middle Eastern/Arab	1 (6.3)
Education, *n*(%)	
High school or equivalent	2 (12.5)
Some college	1 (6.3)
College or university graduate	5 (31.3)
Graduate or professional school	8 (50.0)
Work status, *n*(%)	
Work full-time for pay	4 (25.0)
Work part-time for pay	1 (6.3)
Not working	5 (31.3)
Retired	6 (37.5)
Source of HP *n*(%)	
Post-surgical	13 (81.3)
Idiopathic	2 (12.5)
Genetic	1 (6.3)
Duration of diagnosis (in years)	
Mean (SD)	15.0 (9.9)
Median	12.5
(Range)	(4.0–39.5)
Number of comorbidities	
Mean (SD)	5.1 (3.3)
Median	4.0
(Range)	(1–14)
Most frequently reported medical conditions^a^, *n*(%)	
Hypothyroidism	14 (87.5)
Hypertension	7 (43.8)
Obesity	6 (37.5)
Chronic kidney disease	5 (31.3)
Sleep apnea	5 (31.3)
Stomach or intestinal problems	5 (31.3)
Chronic back pain	4 (25.0)
Kidney stones	4 (25.0)
Reflux disease	4 (25.0)
Current severity of HP symptoms (self-report), *n*(%)	
Very Mild	1 (6.3)
Moderate	12 (75.0)
Between Moderate and Severe	1 (6.3)
Severe	2 (12.5)
Medications and supplements currently used to treat or manage HP^a^, *n*(%)	
Calcium supplements	14 (87.5)
Vitamin D supplements^b^	14 (87.5)
OTC Vitamin D	8 (50.0)
Prescription Vitamin D	11 (68.8)
Forteo	3 (18.8)
Natpara (PTH [1–84])	6 (37.5)
TransCon™ PTH	2 (12.5)
Hydrochlorothiazide (HCTZ)	5 (31.3)
Magnesium supplements	8 (50.0)

^a^Participants were instructed to select all that apply

^b^Number of participants reporting taking Vitamin D as over-the-counter and/or prescription

*HCTZ* hydrochlorothiazide, *HP* hypoparathyroidism, *OTC* over-the-counter, *PTH* parathyroid hormone, *SD* standard deviation

### Comprehension of instructions

Participants were asked if they understood the instructions provided throughout the SF-36v2, as shown in Table 2. All participants confirmed that they clearly understood the primary instructions at the beginning of the survey (Instruction 1), and the instruction pertaining to the Role—Physical and Role—Emotional domains (Instruction 2). In addition, the vast majority further reported that they understood the instructions for the General Health domain (Instruction 4) (*n* = 15, 93.8%). Although a small number of participants (*n* = 3, 18.8%) reported that the instructions for items in the Mental Health and Vitality domains (Instruction 3) were too wordy and/or vague, all were still able to complete the items in that section of the survey. Based on these findings and a review of the participants’ comments on the instructions and corresponding items, it was determined by the research team that issues raised were based on individual preference or style-related changes rather than reflecting a true lack of understanding of the instruction’s intent.

**Table 2:** Instruction comprehension

Instruction, *n*(%) yes	Clearly understood? (*n* = 16)
*Instruction 1: primary instruction.* This survey asks for your views about your health. This information will help keep track of how you feel and how well you are able to do your usual activities. Thank you for completing this survey! For each of the following questions, please select the one response that best describes your answer	16 (100.0)
*Instruction 2: Role—Physical and Role—Emotional domains.* During the past week, how much of the time have you had any of the following problems with your work or other regular daily activities?	16 (100.0)
*Instruction 3: Mental Health and Vitality domains.* This question is about how you feel and how things have been with you during the past week. Please give the one answer that comes closest to the way you have been feeling	13 (81.3)
*Instruction 4: General Health domain.* How TRUE or FALSE is the following statement for you?	15 (93.8)
CD interview guide question regarding instructions	
Were you able to understand and follow the instructions for each section of the survey?	15 (93.8)

Italics indicate primary instruction or instruction used within a domain(s)

### Item comprehension, relevance, importance, and sensitivity to change

Participants confirmed that the items in the SF-36v2 were understandable, relevant, important, and sensitive to change in relation to HP, with nearly all of the questions regarding these aspects being answered affirmatively by the majority of respondents for each item. These findings are shown in Table 3 and summarized further in the subsections below.

**Table 3:** Item comprehension, relevance, importance, and sensitivity to change

Domain/Item, *n*(%) yes	Clearly understood? (*n* = 16)	Relevant to experience with HP? (*n* = 16)	Important aspect of how HP affects functioning and well-being? (*n* = 16)	If relevant, would response change if HP improved or worsened?^a^
*Physical functioning*				
Does your health now limit you in…				
Vigorous activities, such as running, lifting heavy objects, participating in strenuous sports	16 (100.0)	16 (100.0)	14 (87.5)	12/16 (75.0)
Moderate activities, such as moving a table, pushing a vacuum cleaner, bowling, or playing golf	16 (100.0)	16 (100.0)	14 (87.5)	14/16 (87.5)
Lifting or carrying groceries	16 (100.0)	13 (81.3)	12 (75.0)	13/13 (100.0)
Climbing several flights of stairs	16 (100.0)	14 (87.5)	13 (81.3)	12/14 (85.7)
Climbing one flight of stairs	16 (100.0)	13 (81.3)	13 (81.3)	11/13 (84.6)
Bending, kneeling, or stooping	16 (100.0)	10 (62.5)	10 (62.5)	7/10 (70.0)
Walking more than a mile	16 (100.0)	15 (93.8)	13 (81.3)	14/15 (93.3)
Walking several hundred yards	16 (100.0)	14 (87.5)	14 (87.5)	14/14 (100.0)
Walking one hundred yards	16 (100.0)	13 (81.3)	12 (75.0)	12/13 (92.3)
Bathing or dressing yourself	16 (100.0)	11 (68.8)	8 (50.0)	11/11 (100.0)
*Role—physical*				
During the past week, how much of the time have you had any of the following problems with your work or other regular daily activities?				
Cut down on the amount of time you spent on work or other activities as result of your physical health	16 (100.0)	16 (100.0)	15 (93.8)	15/16 (93.8)
Accomplished less than you would like as a result of your physical health	16 (100.0)	16 (100.0)	15 (93.8)	15/16 (93.8)
Were limited in the kind of work or other activities as a result of your physical health	14 (87.5)	14 (87.5)	13 (81.3)	13/14 (92.9)
Had difficulty performing the work or other activities as a result of your physical health (for example, it took extra effort)	16 (100.0)	16 (100.0)	16 (100.0)	16/16 (100.0)
*Bodily pain*				
How much bodily pain have you had during the past week	16 (100.0)	16 (100.0)	14 (87.5)	16/16 (100.0)
During the past week, how much did pain interfere with your normal work (including both work outside the home and housework)	16 (100.0)	15 (93.8)	15 (93.8)	15/15 (100.0)
*General health*				
In general, would you say your health is…	16 (100.0)	16 (100.0)	12 (75.0)	16/16 (100.0)
How TRUE or FALSE is the following statement for you?				
I seem to get sick a little easier than other people	16 (100.0)	10 (62.5)	10 (62.5)	9/10 (90.0)
I am as healthy as anybody I know	16 (100.0)	15 (93.8)	15 (93.8)	11/15 (73.3)
I expect my health to get worse	16 (100.0)	16 (100.0)	16 (100.0)	12/16 (75.0)
My health is excellent	16 (100.0)	13 (81.3)	13 (81.3)	10/13 (76.9)
*Vitality*				
How much of the time during the past week did you…				
Feel full of life	16 (100.0)	15 (93.8)	15 (93.8)	14/15 (93.3)
Have a lot of energy	16 (100.0)	16 (100.0)	16 (100.0)	14/16 (87.5)
Feel worn out	16 (100.0)	16 (100.0)	16 (100.0)	16/16 (100.0)
Feel tired	16 (100.0)	16 (100.0)	15 (93.8)	14/16 (87.5)
*Social functioning*				
During the past week…				
To what extent has your physical health or emotional problems interfered with your normal social activities with family, friends, neighbors, or groups	16 (100.0)	15 (93.8)	15 (93.8)	15/15 (100.0)
How much of the time has your physical health or emotional problems interfered with your social activities (like visiting with friends, relatives, etc.)	16 (100.0)	15 (93.8)	15 (93.8)	15/15 (100.0)
*Role—emotional*				
During the past week, how much of the time have you had any of the following problems with your work or other regular daily activities?				
Cut down on the amount of time you spent on work or other activities as a result of any emotional problems (such as feeling depressed or anxious)	16 (100.0)	16 (100.0)	14 (87.5)	16/16 (100.0)
Accomplished less than you would like as a result of any emotional problems (such as feeling depressed or anxious)	16 (100.0)	14 (87.5)	13 (81.3)	14/14 (100.0)
Did work or other activities less carefully than usual as a result of any emotional problems (such as feeling depressed or anxious)	14 (87.5)	11 (68.8)	10 (62.5)	11/11 (100.0)
*Mental health*				
How much of the time during the past week have you…				
Been very nervous	16 (100.0)	11 (68.8)	10 (62.5)	9/11 (81.8)
Felt so down in the dumps that nothing could cheer you up	16 (100.0)	12 (75.0)	12 (75.0)	11/12 (91.7)
Felt calm and peaceful	16 (100.0)	15 (93.8)	14 (87.5)	15/15 (100.0)
Felt downhearted and depressed	16 (100.0)	16 (100.0)	15 (93.8)	15/16 (93.8)
Been happy	16 (100.0)	14 (87.5)	12 (75.0)	13/14 (92.9)
Change in health status over past week				
Compared to one week ago, how would you rate your health in general now	16 (100.0)	16 (100.0)	13 (81.3)	16/16 (100.0)

^a^Percentages for sensitivity to change are based on the number of participants who reported the item to be relevant to their experience with HP, as indicated by the proportions shown in each row (e.g., 11/12, 15/15, etc.)

#### Item comprehension

Item comprehension was assessed based on participants’ descriptions of what each item meant, how they chose their answer, and whether they reported having difficulty in interpreting the item’s intent. Overall item comprehension of the measure was very high, with 34/36 items being clearly understood by the entire sample, and the remaining 2 items being clearly understood by 87.5% (*n* = 14) of the sample (“Were limited in the kind of work or other activities as a result of your physical health” and “Did work or other activities less carefully than usual as a result of any emotional problems (such as feeling depressed or anxious))”.

#### Relevance and importance

Study participants confirmed that the items in the SF-36v2 were relevant to their experience with HP and represented an important aspect of how HP affected their functioning and well-being. Exemplary quotes are provided in Table 4. Endorsement rates were higher than endorsement rates accepted from concept elicitation interviews as criteria for inclusion of a concept in a newly developed PRO. At least 75% of participants endorsed 31/36 items for both relevance and importance. Of the remaining items, 4 were reported to be both relevant and important by at least 62.5% (*n* = 10) participants (“Bending, kneeling, or stooping”, “I seem to get sick a little easier than other people”, “Did work or other activities less carefully than usual as a result of any emotional problems (such as feeling depressed or anxious), and “Been very nervous”); and 1 was reported to be relevant by 68.8% and important by 50.0% of participants (“Bathing or dressing yourself”).

**Table 4:** Exemplary quotes for selected SF-36v2 items

Domain/item	Exemplary quotes
Physical functioning	
Vigorous activities, such as running, lifting heavy objects, participating in strenuous sports	I answered yes, limited a lot. I did that because I can’t do any of those things, in general. My hypopara specifically, in reference to my health, I don’t run, I can’t lift heavy objects or do strenuous sports anyway, so yes, clearly, I’m limited. (109; female, age 36, works part-time, idiopathic HP)
Role—physical	
Were limited in the kind of work or other activities as a result of your physical health	I said a little of the time…Because I wanted to sit at my desk and do some writing and I couldn’t, because it felt like my legs were falling asleep when I was sitting, so I just laid down. (102; female, age 43, not working, post-surgical HP)
Bodily pain	
During the past week, how much did pain interfere with your normal work (including both work outside the home and housework)?	I would say quite a bit…the past week has been more stable than in some weeks and I did not experience tetany which would definitely change my answer. (116; female, age 54, retired, post-surgical HP)
General health	
In general, would you say your health is…	I took it as being in general my whole health situation. Some people may have more than one problem with their health and some may only have the hypopara situation…I'm looking at my general overall health and that hypopara, if somebody says, “So what are your problems,” hypopara is going to be the first or second answer. (108; female, age 62, retired, post-surgical HP)
Vitality	
Feel full of life	I don’t think that there is any week that I ever feel full of life because of hypopara in general and its effects and side-effects and how it affects my quality of life. I don’t think there is ever a day where I feel full of life. (109; female, age 36, works part-time, idiopathic HP)
Social functioning	
How much of the time has your physical health or emotional problems interfered with your social activities (like visiting with friends, relatives, etc.)	I would say most of the time, just because…when you have anxiety and depression, and just hypopara in general, you're managing your calcium levels, you tend to avoid a lot of friends and kind of stick to yourself like an introvert. (101; male, age 29, works full-time, idiopathic HP)
Role—emotional	
Accomplished less than you would like as a result of any emotional problems (such as feeling depressed or anxious)	Participant: Most of the time, again, the anxiety is so severe that it makes me where I just struggle to successfully do activities or complete tasks that needs to be done. Interviewer: Is this question relevant to your hypopara? Participant: Yes. (115; female, age 41, not working, post-surgical HP)
Mental health	
Felt so down in the dumps that nothing could cheer you up	I put a little of the time…So, yes, the depression that comes with the hypopara, too. (103; male, age 62, retired, post-surgical HP)

#### Sensitivity to change

Study participants further confirmed that the items in the SF-36v2 were sensitive to changes in their condition. Of those who had endorsed an item as being relevant to their experience with HP, at least 75.0% further reported that the response option they had chosen would change if their condition improved or worsened for 34/36 items; the remaining 2 items had endorsement rates of at least 70.0% (“Bending, kneeling, or stooping” and “I am as healthy as anybody I know”).

### Response option comprehension

The entire study sample confirmed that they understood all of the response options throughout the measure and that each set of response options was appropriate for its corresponding items. All suggested changes to the response options were determined to be based on individual preferences (e.g., to add, remove, or change wording of response options) rather than issues with being able to interpret their meaning.

### Frequency distribution of item responses

The frequency distribution of participant responses to each item was reviewed for potential floor and ceiling effects, as shown in Table 5. In accordance with other studies, floor effects were assessed based on the percentage of participants choosing worst possible response option (reflecting worst health state) for a given item, and ceiling effects were assessed based on the percentage choosing the best possible response option (reflecting the best health state) [19–21]. For this study, floor and ceiling effects were defined as 50% or greater selected either the highest or lowest response option for an item.

**Table 5:** Frequency distribution of item responses

		Total (*n* = 16)	
Domain/item	Range of responses chosen	Floor, *n* (%)	Ceiling, *n* (%)
*Physical functioning*			
Does your health now limit you in…			
Vigorous activities, such as running, lifting heavy objects, participating in strenuous sports	Yes, limited a lot to Yes, limited a little	8 (50.0)	0 (0)
Moderate activities, such as moving a table, pushing a vacuum cleaner, bowling, or playing golf	Yes, limited a lot to No, not limited at all	8 (50.0)	3 (18.8)
Lifting or carrying groceries	Yes, limited a lot to No, not limited at all	2 (12.5)	9 (56.3)
Climbing several flights of stairs	Yes, limited a lot to No, not limited at all	8 (50.0)	4 (25.0)
Climbing one flight of stairs	Yes, limited a lot to No, not limited at all	2 (12.5)	6 (37.5)
Bending, kneeling, or stooping	Yes, limited a lot to No, not limited at all	3 (18.8)	8 (50.0)
Walking more than a mile	Yes, limited a lot to No, not limited at all	9 (56.3)	4 (25.0)
Walking several hundred yards	Yes, limited a lot to No, not limited at all	3 (18.8)	8 (50.0)
Walking one hundred yards	Yes, limited a lot to No, not limited at all	2 (12.5)	12 (75.0)
Bathing or dressing yourself	Yes, limited a little to No, not limited at all	0 (0)	11 (68.8)
*Role—physical*			
During the past week, how much of the time have you had any of the following problems with your work or other regular daily activities?			
Cut down on the amount of time you spent on work or other activities as result of your physical health	All of the time to A little of the time	1 (6.3)	0 (0)
Accomplished less than you would like as a result of your physical health	All of the time to None of the time	4 (25.0)	1 (6.3)
Were limited in the kind of work or other activities as a result of your physical health	All of the time to None of the time	4 (25.0)	2 (12.5)
Had difficulty performing the work or other activities as a result of your physical health (for example, it took extra effort)	All of the time to A little of the time	1 (6.3)	0 (0)
*Bodily pain*			
How much bodily pain have you had during the past week	Very severe to Very mild	2 (12.5)	0 (0)
During the past week, how much did pain interfere with your normal work (including both work outside the home and housework)	Extremely to Not at all	2 (12.5)	2 (12.5)
*General health*			
In general, would you say your health is…	Poor to Excellent	1 (6.3)	1 (6.3)
How TRUE or FALSE is the following statement for you?			
I seem to get sick a little easier than other people	Definitely true to Definitely false	5 (31.3)	3 (18.8)
I am as healthy as anybody I know	Definitely false to Definitely true	8 (50.0)	1 (6.3)
I expect my health to get worse	Definitely true to Mostly false	2 (12.5)	0 (0)
My health is excellent	Definitely false to Definitely true	9 (56.3)	1 (6.3)
*Vitality*			
How much of the time during the past week did you…			
Feel full of life	None of the time to Most of the time	3 (18.8)	0 (0)
Have a lot of energy	None of the time to Most of the time	4 (25.0)	0 (0)
Feel worn out	All of the time to Some of the time	2 (12.5)	0 (0)
Feel tired	All of the time to Some of the time	5 (31.3)	0 (0)
*Social functioning*			
During the past week…			
To what extent has your physical health or emotional problems interfered with your normal social activities with family, friends, neighbors, or groups	Quite a bit to Not at all	0 (0)	2 (12.5)
How much of the time has your physical health or emotional problems interfered with your social activities (like visiting with friends, relatives, etc.)	All of the time to None of the time	2 (12.5)	2 (12.5)
*Role—emotional*			
During the past week, how much of the time have you had any of the following problems with your work or other regular daily activities?			
Cut down on the amount of time you spent on work or other activities as a result of any emotional problems (such as feeling depressed or anxious)	Most of the time to None of the time	0 (0)	4 (25.0)
Accomplished less than you would like as a result of any emotional problems (such as feeling depressed or anxious)	Most of the time to None of the time	0 (0)	6 (37.5)
Did work or other activities less carefully than usual as a result of any emotional problems (such as feeling depressed or anxious)	Most of the time to None of the time	0 (0)	8 (50.0)
*Mental health*			
How much of the time during the past week have you…			
Been very nervous	All of the time to None of the time	1 (6.3)	4 (25.0)
Felt so down in the dumps that nothing could cheer you up	Some of the time to None of the time	0 (0)	8 (50.0)
Felt calm and peaceful	Most of the time to None of the time	2 (12.5)	0 (0)
Felt downhearted and depressed	Some of the time to None of the time	0 (0)	8 (50.0)
Been happy	A little of the time to Most of the time	0 (0)	0 (0)
Change in health status over past week			
Compared to one week ago, how would you rate your health in general now	About the same to Much better	1 (6.3)	0 (0)

Floor effects were observed in 6 items: 4 in the physical functioning domain, and 2 in the general health domain. These effects were likely due to all but 1 participant having self-reported the current severity of their HP symptoms as either moderate or severe, and the majority having reported that they found it “a lot” or “extremely” difficult to manage their HP.

Ceiling effects were observed in 8 items: 5 in the physical functioning domain, 1 in the Role—Emotional domain, and 2 in the Mental Health domain. However, most participants considered each of these items to be relevant (62.5%–100.0%) and/or important (50.0%–93.8%) to their condition, and the vast majority (70.0%–100.0%) of participants who reported an item to be relevant to their experience with HP further indicated that their responses would change if their condition either improved or worsened. Therefore, the content should be considered relevant.

### Recall period

For survey items with a 1-week recall period, participants were asked whether they felt that this timeframe was appropriate considering what the questions were about, and whether they were able to accurately remember their experiences over this time frame. Most participants (*n* = 15, 93.8%) were able to accurately recall their experiences over the past week (data missing for 1 participant).

Just under half of participants (*n* = 7, 43.8%) considered a 1-week recall period to be optimal. Nine participants, including 2 who considered the time frame to be acceptable, felt that a longer time frame would be more appropriate given the variations they have experienced in the symptoms and impacts associated with HP over time. One participant (6.3%) felt that the time frame was too short because her symptoms tended to vary on a daily basis. It should be noted that the alternative version of the SF-36v2 asks about a 1-month recall which we believe would have been too long for this population given HP-related cognitive issues. Therefore, the acute SF-36v2 1-week recall is preferred.

## Discussion

The study findings show that the items in the SF-36v2 are applicable to adults with chronic HP. All items in the SF-36v2 were reported to be understood, relevant, important, and sensitive to change by at least half, and in most cases, a strong majority of study participants. Overall, item comprehension of the measure was very high, with 34/36 items being clearly understood by the entire sample, and the remaining 2 items being clearly understood by 87.5% (*n* = 14) of the sample. At least 75% of participants endorsed 31/36 items for both relevance and importance. Of the remaining items, 4 were reported to be both relevant and important by at least 62.5% (*n* = 10) participants and 1 was reported to be relevant by 68.8% and important by 50.0% of participants. Of those who had endorsed an item as being relevant to their experience with HP, at least 75.0% further reported that the response option they had chosen would change if their condition improved or worsened for 34/36 items; the remaining 2 items had endorsement rates of at least 70.0%.

Most participants understood clearly the instructions provided throughout the SF-36v2, and the entire study sample confirmed that they understood all of the response options throughout the measure and found them appropriate. The majority of participants (*n* = 15, 93.8%) were able to accurately recall their experiences over the past week (data missing for one participant). Although some participants (*n* = 9, 56.3%) indicated they thought a longer recall period would be more appropriate, the acute SF-36v2 1-week recall is preferred by the research team over the 1-month recall of the alternative version of the SF-36v2 due to the 1-month time frame being too long for this population given HP-related cognitive issues.

Floor effects were observed in 6 items and were likely due to all but one participant having a disease severity of either moderate or severe, and the majority having reported that they found it “a lot” or “extremely” difficult to manage their HP. Ceiling effects were observed in 8 items; however, most participants considered each of these items to be relevant (62.5%–100%) and/or important (50%–93.8%) to their condition, and the vast majority (70.0%–100.0%) further reported that their responses would change if their condition either improved or worsened. Therefore, the content should be considered relevant.

The study findings are consistent with what would be expected; not every item would be relevant or important for every person with HP, just as, for any other measure, not everyone experiences every symptom or impact. The overall high levels of endorsement provide strong evidence of the measure’s content validity for this population with respect to the symptoms and impacts that it is intended to measure. Endorsement rates were higher than accepted endorsement rates from concept elicitation interviews for inclusion of a concept in a newly developed PRO. Additionally, study participants confirmed that the items in the SF-36v2 were sensitive to changes in their condition, which is an essential component when endorsing a measure for use in clinical trials.

Since development of the SF-36 in 1988, the family of SF-36 measures has become one of the most well used, generic health status assessment measures currently available [15]. It was originally developed on a diverse disease patient population without individual conditions being analyzed as subgroups. Since its creation, the SF-36 family of measures has been used in studies in multiple conditions. However, the content validity of the measure has not been examined, with some exceptions such as including diabetes, AL amyloidosis, and lupus [16–18]. Unfortunately, it is often incorporated into research without first confirming that the measure is suitable and has content validity for the specific population under study. In HP research, as with other conditions, it has often been used as a generic measure in both clinical trials and health outcomes research in the US as well as globally [22–28]. This study is the first to examine and confirm that the SF-36v2 is in fact suitable for use in an HP population.

As with all studies, there are limitations. This study was conducted on a US sample and generalizability to other countries and cultures may also need to be examined. Also, given that the SF-36v2 is a generic measure, which may not cover all of the major symptoms and impacts associated with this condition, and has a 1-week recall, which may not capture all the fluctuations of the disease, it is also recommended that its usage be supplemented with disease-specific instruments such as the recently developed Hypoparathyroidism Patient Experience Scale—Symptom (HPES-Symptom) and Hypoparathyroidism Patient Experience Scale—Impact (HPES-Impact) measures [29–31].

The study findings show that the items in the SF-36v2 are applicable and relevant to adults with chronic HP and that the items represented an important aspect of how HP affected their functioning and well-being. Participants confirmed that the items in the SF-36v2 were understandable, relevant, important, and sensitive to change in relation to HP, with strong majorities answering affirmatively to nearly all of these questions for every item. The SF-36v2 is therefore recommended for usage in clinical trials examining adults with HP.

## Data Availability

The data for the research presented in the publication may be available on a case-by-case basis for reasonable requests from the corresponding author.
